# Ultrahigh field MR imaging of a subconjunctival anti-glaucoma drug delivery system in a rabbit model

**DOI:** 10.1038/s41598-017-15954-w

**Published:** 2017-11-17

**Authors:** Franziska Kopp, Thomas Eickner, Stefan Polei, Karen Falke, Martin Witt, Niels Grabow, Oliver Stachs, Rudolf F. Guthoff, Tobias Lindner

**Affiliations:** 1 Department of Ophthalmology, Rostock University Medical Center, Rostock, Germany; 2 Institute for Biomedical Engineering, Rostock University Medical Center, Rostock, Germany; 3 Core Facility Multimodal Small Animal Imaging, Rostock University Medical Center, Rostock, Germany; 4 Institute for Anatomy, Rostock University Medical Center, Rostock, Germany

## Abstract

Local drug delivery systems (DDS) have become a favourable approach for the treatment of numerous diseases. Biomedical imaging techniques such as ultrahigh field magnetic resonance imaging (UHF-MRI) offer unique insight into DDS biodegradation *in vivo*. We describe the establishment of a 7 Tesla MRI routine for longitudinal *in vivo* examinations of a subconjunctival DDS for the treatment of glaucoma in a rabbit model. In initial *in vitro* examinations the T2-relaxation times of the polymeric DDS components were assessed. Imaging of enzymatically degraded depot samples *in vitro* did not reveal changes in sample morphology or T2-relaxation time. *Ex vivo* investigations with an enucleated porcine eye showed good correlation of anatomical MRI and histological data. In longitudinal *in vivo* studies in rabbits, we repeatedly scanned the depot in the same animal over the course of 5 months with an in-plane resolution of 130 µm at scan times of less than 30 minutes. The degradation was quantified using volumetric analysis showing a volume reduction of 82% between 3 and 21 weeks after depot implantation. We have thereby demonstrated the feasibility of our UHF-MRI protocol as a non-invasive imaging routine for qualitative and quantitative, longitudinal evaluation of biodegradable subconjunctival DDS.

## Introduction

Glaucoma encompasses a class of optic neuropathies characterised by progressive degeneration of retinal ganglion cells and concomitant irreversible vision loss. In 2013, an estimated number of 64.3 million people (ages 40–80) were affected globally, with a projected increase of 74% by 2040^1^. It is the leading cause of irreversible blindness, worldwide. Among the several glaucoma subtypes primary open-angle glaucoma (POAG) has the highest prevalence, accounting for more than 86% of all patients^1^. The leading risk factor associated with POAG is elevated intraocular pressure (IOP)^2^. Reduction of IOP, drug-mediated or surgically, is the only course of treatment which has been proven effective^3^. Hypotensive eye drops are always the first-line therapy, but topical treatment is often inefficient due to poor patient adherence^4^. There is growing evidence that lack of adherence correlates with greater IOP fluctuations and an increase in visual field defects^5–8^. Several strategies are currently investigated in an attempt to relieve patients of treatment regimens that sometimes entail multiple doses of one or more medications a day. Novel drug delivery devices like punctum plugs, inserts, and intraocular implants provide sustained drug release over an extended period of time. They do not rely on daily patient participation for treatment success and are heavily pursued in medical research and development^9–11^.

We recently developed a subconjunctival, *in situ* polymerising, biodegradable drug delivery system (DDS) eluting latanoprost for the treatment of POAG. In a previous *in vivo* study in rabbits we used a surgical microscope for longitudinal monitoring of biodegradation and biocompatibility of the DDS^12^. This method was limited to imaging the surface of the drug depot and did not allow quantification of the degradation process. Yet, successful clinical implementation of periocular DDS requires in-depth knowledge regarding implant position, dimension, and degradation. The latter two being key factors in drug release kinetics^13^. To optimise our DDS evaluation procedure we sought to implement a non-invasive imaging tool to investigate the drug depot *in vivo*.

The eye is generally well suited for magnetic resonance imaging (MRI) because of the high variation in water content of the different ocular structures. Due to the excellent in-plane resolution ultrahigh field MRI (UHF-MRI) has become an important tool in medical research to visualise ocular structures in animals and humans *ex vivo* and *in vivo* ^14–16^.

The present study was conducted to establish an UHF-MRI routine for longitudinal *in vivo* examinations of periocular, biodegradable DDS in rabbits. We initially investigated the MR properties of the DDS components *in vitro*. Subsequently, we studied enzymatic degradation of the polymeric matrix *in vitro* using micro-computed tomography (micro-CT) imaging and scanning electron microscopy (SEM) in combination with UHF-MRI. In the next step, we assessed imaging quality of the drug depot in ocular tissues *ex vivo* using an enucleated porcine eye. Finally, to test the developed imaging routine in longitudinal *in vivo* biodegradation studies, New Zealand White (NZW) rabbits received unilateral subconjunctival injections of the DDS. *In vivo* MRI measurements were performed in combination with biomicroscopic documentation over the course of 21 weeks following the injection.

## Methods

### Drug Delivery System

The biodegradable DDS under investigation consists of a bipartite, polymeric matrix and the hypotensive prodrug latanoprost (Hangzhou Dayangchem Co. Ltd., Hangzhou City, China). The matrix is a 1/1 (v/v) mixture of the biopolymer hyaluronic acid (HA) and the proprietary cross-linking agent ELA-NCO, an isocyanate-functionalised 1,2-ethylene glycol bisdilactic acid derivate. ELA-NCO was formulated according to Sternberg and colleagues^17^, and subsequently diluted with 15% DMSO (Sigma-Aldrich, Taufkirchen, Germany) to reduce viscosity. Hyaluronic acid sodium salt from *Streptococcus equi* (Sigma-Aldrich, Taufkirchen, Germany), with a molecular weight of 1.5–1.8 MDa, was prepared with latanoprost (HA(LP)), as described previously^12^.

### *In vitro* polymerisation and degradation experiments

For *in vitro* polymerisation both components were transferred to a double chamber syringe equipped with a single screw extruder (Medmix, Risch-Rotkreuz, Switzerland). Polymerisation was initiated by extrusion mixing of the components into a 2 ml Eppendorf tube.

MR imaging of the syringe containing the separate components, and the Eppendorf tube with the polymerised sample, 10 minutes and 16 hours after mixing, was performed with an ultrahigh field small animal MR scanner (7 Tesla, BioSpec 70/30 USR, gradient: B-GA12S, gradient strength: 440 mT/m, Bruker BioSpin MRI GmbH, Ettlingen, Germany) equipped with an 86 mm volume resonator transmit-only coil, and a 2x2 receive-only surface coil (both Bruker). Specimens were imaged using a T2-weighted (T2w) TurboRARE sequence (TE/TR = 35/2500 ms, rare factor = 8, averages = 3, FoV = 20 × 18.7 mm, matrix = 133 × 125, slice thickness = 1.0 mm, in-plane resolution = 150 µm × 150 µm). The signal intensity was plotted, and the area under the curve (AUC) calculated with Prism 6.07 (Graph Pad Software Inc., La Jolla, USA).

For *in vitro* degradation samples were prepared as previously described^12^. In brief, several specimens of the HA/ELA-NCO matrix with equal shape and size were fabricated using Teflon moulds, weighed, and incubated in Sørensen buffer, containing 1.5 µg/mL lysozyme, over the course of several weeks (2, 4, 12, 20, and 50). The degradation was stopped by washing the specimens with deionised water after which they were dried in a desiccator at room temperature and weighed again.

For MR imaging the specimens were placed in an Eppendorf tube (1.5 ml), rehydrated in Sørensen buffer without lysozyme, and subjected to vacuum to remove all remaining air from sample cavities. MR images were acquired with an MRI scanner equipped with a ^1^H Quadrature transmit/receive cryogenic RF coil (CryoProbe, Bruker). The specimens were imaged using a T2w TurboRARE sequence (TE/TR = 35/2500 ms, rare factor = 8, averages = 7, FoV = 8.4 × 9.5 mm, matrix = 84 × 96, slice thickness = 0.5 mm, in-plane resolution = 100 µm × 100 µm) for anatomical reference, and with a multi-slice, multi-echo sequence (MSME, TR: 2000 ms, TE: 10 echoes equally distributed between 7.79 to 77.95 ms, averages 15, FoV = 8.4 × 8.6 mm, matrix = 56 × 57, slice thickness = 0.5 mm, in-plane resolution = 150 × 150 µm) for T2-relaxation time mapping. T2-relaxation maps were calculated using the fitting routine implemented in Bruker Paravision software v.6.01. Statistical analysis of T2-relaxation times was carried out using one-way analysis of variance with Dunnett’s post-test (Prism 6.07).

The micro-CT investigations were performed using a high resolution x-ray micro-computed tomography scanner (SkyScan1172, SkyScan N.V., Aartselaar, Belgium) equipped with an 80 kV microfocus X-ray source (Hamamatsu Photonics K.K., Iwata-City Shizuoka, Japan). Images were acquired with the following parameters: voltage = 80 kV, electron current = 100 μA, pixel size = 7.3 μm, rotation range = 180°, rotation step = 0.7°, no filter.

The surface morphology of the degradation samples was investigated using scanning electron microscopy with the Quanta FEG 250 field emission scanning electron microscope with a large field detector (FEI, Graefelfing, Germany). Samples were fixed on trays with conductive tape and secondary electron micrographs were taken with spot size 3 in low vacuum mode at 0.5 mbar and 3 kV.

### *Ex vivo* experiments with a porcine eye

Assessment of image quality in ocular tissues was done *ex vivo* using a porcine eye (German Landrace swine). To compare high resolution morphological data from *in situ* MRI measurements and histology analysis, the DDS was injected into the subconjunctival space of an enucleated pig eye with the above described syringe and extruder in combination with a 27 G needle. MR imaging was performed 90 minutes after depot injection. For anatomical MR imaging the B-GA12S gradient, the 2 × 2 array surface coil, and the 86 mm transmit volume coil were used. Parameters for T2w TurboRARE sequence were as follows: TE/TR = 42/4625 ms, rare factor = 8, averages = 5, FoV = 31.4 × 28.3 mm, matrix = 313 × 282, slice thickness = 0.75 mm, in-plane resolution = 100 µm × 100 µm.

Following MRI the eye was prepared for histological analysis by fixation in a 4% formaldehyde solution (Formafix, Grimm GmbH, Germany) at 4 °C for 48 hours. It was dissected along the sagittal plane into two halves, dehydrated, and embedded in paraffin. The specimens were then cut perpendicular to the iris in series of 5 μm sections. After dewaxing in xylene, sections were rehydrated in a graded series of ethanol, stained with haematoxylin and eosin (H&E), dehydrated, cleared in xylene, and mounted in DePeX (Serva, Heidelberg, Germany).

### *In vivo* degradation experiments in rabbits

For longitudinal *in vivo* investigations, the DDS was injected into the subconjunctival space of three female New Zealand White rabbits (Charles River GmbH, Sulzfeld, Germany). The injection was carried out as described above under general anaesthesia, with 50 mg/kg ketamine hydrochloride (Bela-pharm GmbH & Co. KG, Vechta, Germany) and 5 mg/kg xylazine hydrochloride (Rompun; Bayer Health Care, Leverkusen, Germany). At 3, 12, and 21 weeks after injection, biomicroscopic documentation and MR imaging of the drug delivery system were performed, also under general anaesthesia. The MRI scanner was setup with a B-GA20S gradient (gradient strength: 220 mT/m) and ^1^H transmit coil with an inner diameter of 154 mm and a 2x2 receive-only surface coil. The animals were placed in left lateral recumbent position. To prevent the eyes from drying during the scan procedure they were treated with Vidisic eye gel (Bausch & Lomb GmbH, Berlin, Germany) and the lids of the injected eye were carefully shut with surgical tape. The surface coil was placed directly over the lid of the injected eye and fixed in place with adhesive tape. During the first measurements a water filled circular tube was placed between eye and coil, for orientation purposes. Scan parameters for T2w TurboRARE sequences are summarised in Table 1. Volumetric segmentation in the sagittal plane and analysis of the drug depot was carried out using ITK-Snap^18^. After the final examination the animal was sacrificed.

**Table 1:** MR scan parameters of T2w sequences used for *in vivo* imaging of rabbits.

	sagittal	coronal
TE [ms]	38	39
TR [ms]	3,600	3,800
in–plane resolution [µm]	120 × 120	130 × 130
slice thickness [mm]	1.0	1.0
FoV [mm]	34.6 × 39.8	37.15 × 40.15
matrix size	280 × 333	285 × 308
scan time [min]	15–30	15–30

All animal studies were approved by the local authorities (LALLF M-V) and conducted in accordance with the German Animal Welfare Act (Approval ID: 7221.3–1.1–100/11).

The datasets generated and analysed during the current study are available from the corresponding author on reasonable request.

## Results

### MR imaging of the drug delivery system *in vitro*

**Figure 1:**
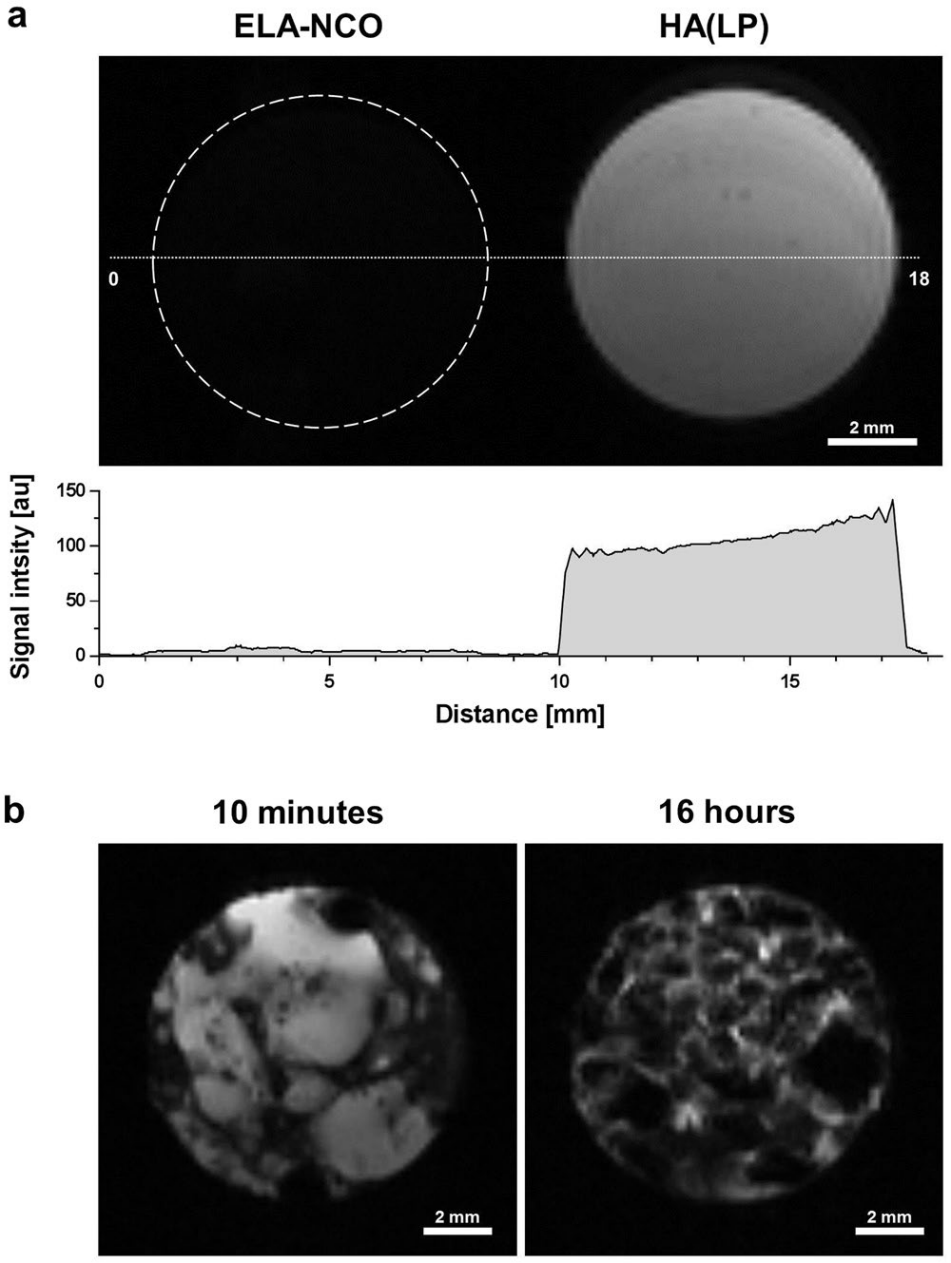
*In vitro* imaging of the DDS. (**a**) Cross section of a double chamber syringe containing the DDS components ELA-NCO and HA(LP). The white dashed circle represents the outline of the ELA-NCO containing chamber. The white dotted line corresponds to the signal intensity plot. (**b**) MR images of the drug depot in an Eppendorf tube, acquired 10 minutes and 16 hours after initiating polymerisation, by mixing the components.

MR imaging of the DDS was performed prior to mixing the components, and after polymerisation. Fig. 1 shows a cross section of a double chamber syringe loaded with ELA-NCO and HA(LP). The cross-linker ELA-NCO presented as hypointense while hyaluronic acid with latanoprost appeared hyperintense with some darker spots. The signal intensity plot demonstrates that hardly any signal was detectable from the cross-linker ELA-NCO in the T2w TurboRARE sequence with the parameters used for imaging (AUC_ELA-NCO_ = 38.26; AUC_HA(LP)_ = 790.3). T2-relaxation times of HA(LP) and ELA-NCO were determined as an average over the depicted cross sections (T2_HA(LP)_ = 296 ms and T2_ELA-NCO_ = 19.7 ms). Fig. 1 shows images acquired 10 minutes after mixing the components and after almost complete polymerisation, 16 hours later. The drug depot presented as a heterogeneous mix of hypointense and hyperintense areas with the latter prevailing early in the polymerisation process. After 16 hours appearance of the specimen had changed considerably. The initially coarse distribution of hyper- and hypointense areas had changed to a finer net-like structure with more dominant hypointense regions.

**Figure 2:**
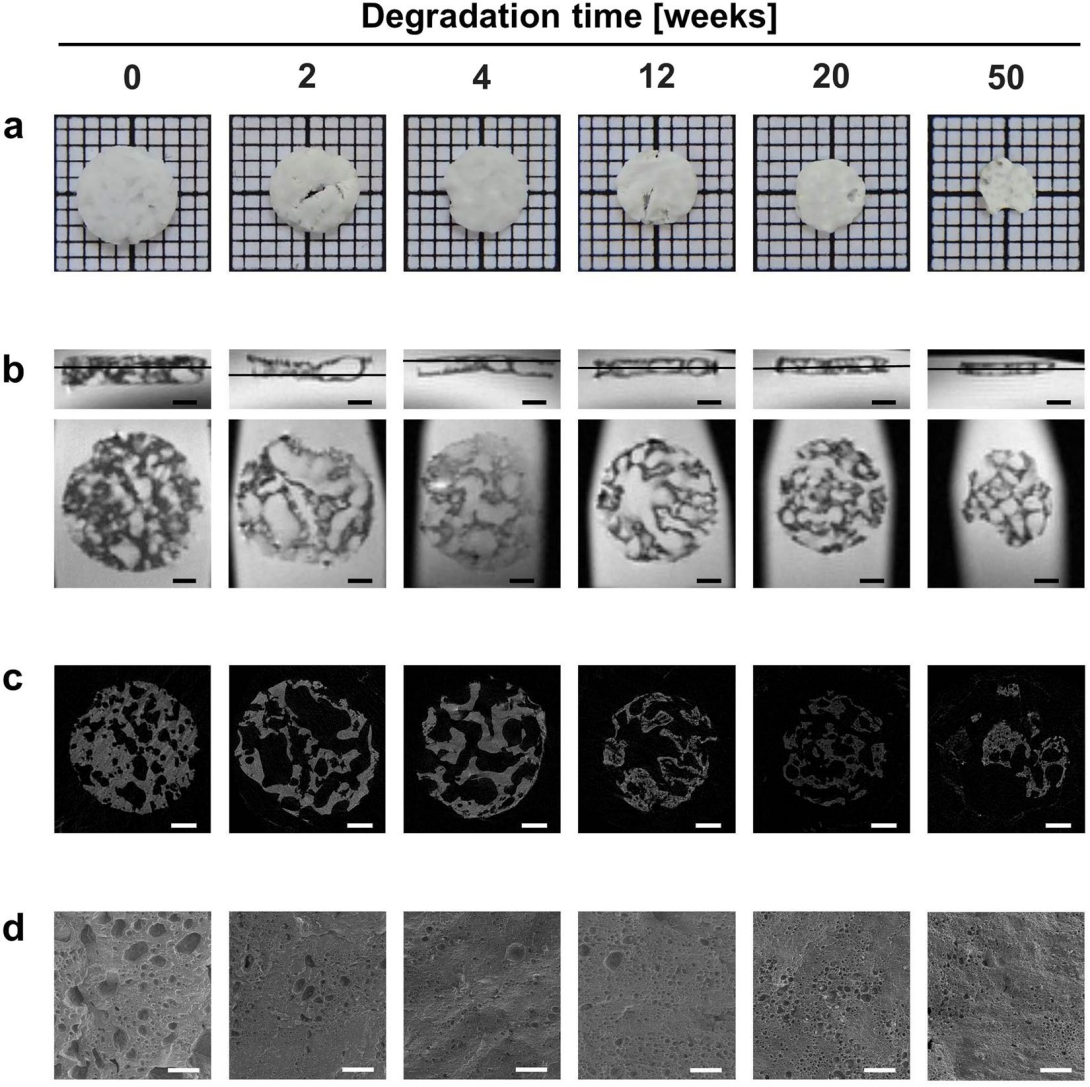
*In vitro* degradation of the DDS matrix. (**a**) Photographs of the degradation specimens used for MRI, micro-CT, and SEM visualisation taken prior to rehydration in buffer. (**b**) MR images acquired with T2w TurboRARE sequences in sagittal (top) and axial direction (bottom). In top images ── marks the respective sections shown in the bottom panel. (**c**) Micro-CT images corresponding to the MRI sections shown in **(b)**. The 50 week sample broke during preparations for micro-CT analysis and only fragments could be imaged. (**d**) High resolution SEM images of the sample surfaces. The scale bar in all images in (**b**) and (**c**) represents 1 mm, and 20 µm in all images in (**d**).

Enzymatic degradation of the polymeric matrix *in vitro* resulted in a decrease of weight (Table 2) and diameter as well as the appearance of cracks and holes in the surface of the specimens (Fig. 2a). The change in diameter from 6 to 4 mm and in height from 1.25 to 0.5 mm was confirmed by MR imaging. In general the samples presented with an irregular texture comprised of hypointense and hyperintense areas of varying sizes (Fig. 2b). The T2-relaxation time of each sample was calculated as the mean of two cross sections and was not affected by the degradation process (Table 2). Statistical analysis revealed no significant differences between sample means (p = 0.84). The hyperintense regions of the MR images correlate well with the dark areas in micro-CT images in all of the degraded samples, corresponding to sample cavities filled with buffer or air, respectively (Fig. 2b,c). SEM images show no profound change in surface morphology between 2 and 50 weeks of degradation (Fig. 2d).

**Table 2:** Weight and T2-relaxation time of *in vitro* degraded samples.

degradation time [weeks]	mass reduction [%]	T2-relaxation time [mean ± s.d. in ms]
0	—	127.9 ± 0.16
2	48.5	132.3 ± 9.7
4	43.6	130.7 ± 0.88
12	60.7	121.8 ± 5.71
20	69.0	127.4 ± 29.5
50	86.8	115.5 ± 14.32

T2-relaxation times shown were calculated as mean of two sections per sample. Individual T2-relaxations times of each section were averaged over the whole T2-map.

### *Ex vivo* imaging of a porcine eye

**Figure 3:**
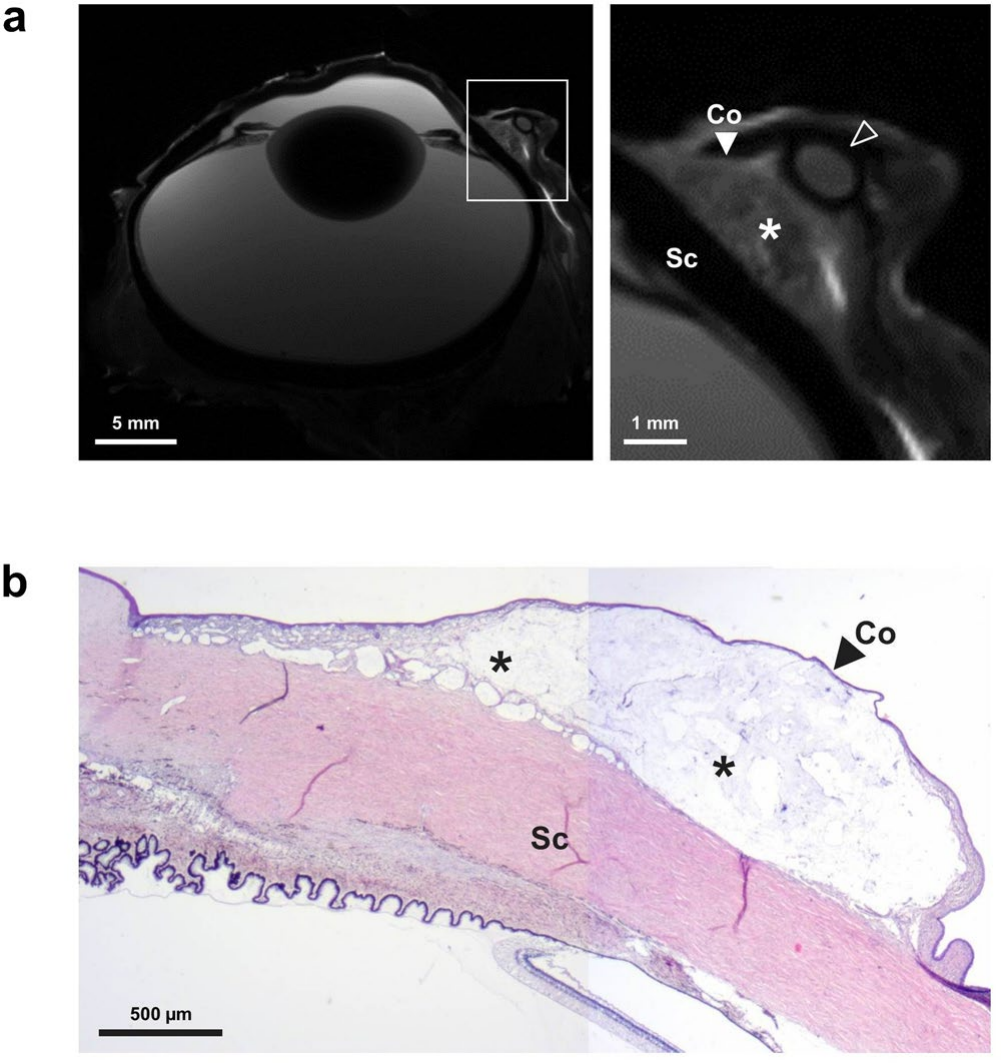
MR imaging and histology of porcine eye with subconjunctival DDS. (**a**) Images of porcine eye acquired using T2w TurboRARE sequence in the axial plane. The drug depot (*) is visible between sclera (Sc) and conjunctiva (Co) as a heterogeneous structure of hyperintense and hypointense regions. The elliptical structure on top of the drug depot seen in the enlargement is part of the nictitating membrane (clear arrowhead), which was removed prior to histological analyses. (**b**) Composite image of two H&E stained sections of the porcine eye, showing the drug depot (*) as a hyaline mass located between conjunctiva (Co) and sclera (Sc).

*Ex vivo* MRI resulted in high quality images of the drug depot with an in-plane resolution of 100 × 100 µm (Fig. 3a). The depot was located close to the limbus, directly above the sclera. The enlargement shows an irregular distribution of hypointense and hyperintense areas within the implant. Haematoxylin and eosin (H&E) staining confirmed the localisation showing a hyaline mass between conjunctiva and sclera (Fig. 3b).

### *In vivo* imaging and biodegradation

**Figure 4:**
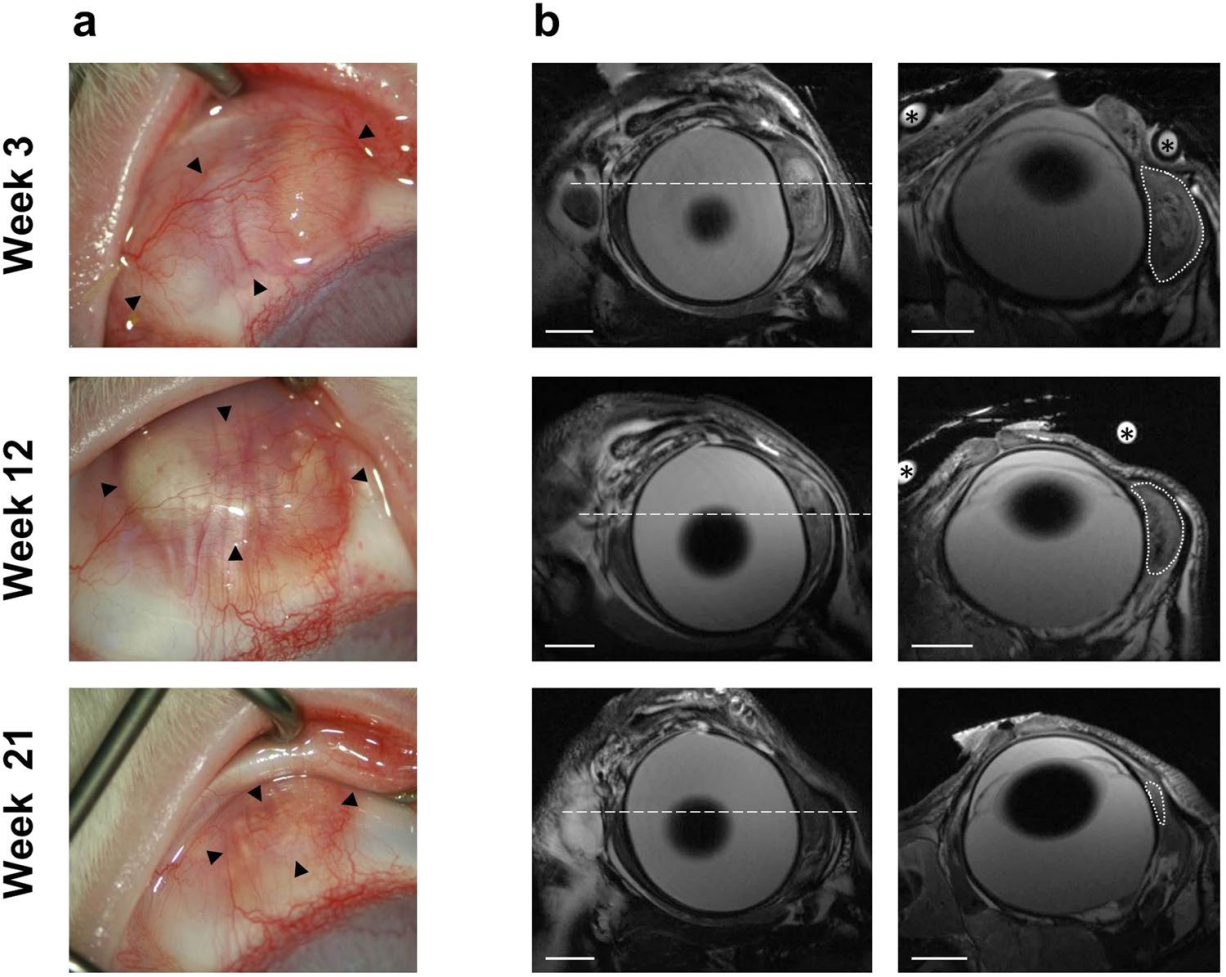
*In vivo* imaging of the DDS during longitudinal rabbit study. (**a**) *In vivo* biomicroscopy photographs showing volume reduction of the DDS (►) over the course of 21 weeks. (**b)** *In vivo* high resolution MR images (coronal - left; sagittal - right), acquired on the same days as the photographs in (**a**). In coronal images ---- marks the respective sagittal sections. In sagittal images ⋅⋅⋅⋅⋅ traces the contour of the drug depot. Cross sections through a water filled circular tube that was used for orientation purposes are marked with *. The scale bar in all MR images represents 5 mm.

*In vivo* MR imaging was performed after application of the DDS into the subconjunctival space of the right eye of three rabbits. No complications occurred during the procedure and only moderate hyperaemia ensued. Following the injection the degradation process was monitored for 5 months. In weeks 3, 12, and 21 after application MRI of the treated eye was performed in combination with biomicroscopy using a surgical microscope. Images of one animal, which received an injection of 77 mg DDS, are shown exemplarily in Fig. 4. Three weeks after the injection, the depot presented as a uniform mass of yellow-ochre coloration forming a ridge stretched between limbus and fornix. In week 12 the depot appeared bifurcated by the superior rectus muscle with a prominent portion on the nasal side of the muscle, and the distal portion showing beginning fragmentation. After 21 weeks biodegradation had progressed so far that the depot mass was fully fragmented (Fig. 4a). T2w MRI sequences acquired were adjusted for optimal contrast between subconjunctival tissue and the drug depot. The resulting images (Fig. 4b) show the delivery system easily distinguishable from surrounding ocular tissues at 3 and 12 weeks after injection. The interior of the depot is heterogeneous with an irregular distribution of hypointense and hyperintense areas. After 21 weeks the drug depot is still visible in MR images, but less distinguishable from surrounding tissues. Quantification of the *in vivo* degradation process using volumetric analysis revealed an 82% reduction over the course of 4.5 months. The depot dimension decreased from 91 mm³ at 3 weeks to 58 mm³ and 16 mm³ at 12 and 21 weeks, respectively.

## Discussion

In the process of developing a subconjunctival DDS for glaucoma treatment, detailed information about biocompatibility, biodegradation, and drug release is essential. Tissue specific enzymatic and cellular processes are largely responsible for biodegradation and consequentially also drug release kinetics. These complex mechanisms can to date not be fully mimicked in *in vitro* settings. Therefore, data gathered from *in vitro* studies has to be evaluated and verified by *in vivo* investigations. Biomedical imaging techniques offer the possibility of long-term, non-invasive *in vivo* analysis of local drug depots in their target tissue^19^.

In previous studies we assessed biodegradation *in vivo* via photo documentation using a surgical microscope^12^. This allowed only superficial evaluation and did not enable quantification of the degradation process. To improve DDS evaluation procedures, we established an imaging protocol using MRI, which allows analysis of implant localisation and long-term monitoring of depot degradation. Standard non-invasive imaging techniques such as OCT and UBM were not applicable due to their physical limitations: OCT has a penetration depth and absorption characteristics insufficient to image a depot with an initial height of 3 to 4 mm after injection, together with underlying ocular structures^20^. While UBM could achieve sufficient penetration depths and resolutions at ultrahigh frequencies, imaging is prone to artefacts and distortion based on scattering, acoustic shadows, and the tissue/material dependent speed of sound^21^. Neither technique would allow high quality imaging of structures behind the fornix. As the depot had relocated to the posterior portion of the eye in some animals in previous studies, we were specifically interested in a technique that enabled imaging of the depot in similar cases.

Magnetic resonance imaging is a well-established tool in clinical routine as well as in ophthalmic research for *ex vivo* and *in vivo* investigations^14–16^. While 1.5 Tesla systems dominate in clinical settings, research has progressed to MRI at high (3 Tesla) and ultrahigh field strengths of 7 Tesla and above^22^. We have previously used a 7 Tesla small animal MR scanner to successfully visualise the anatomical position of experimental suprachoroidal glaucoma drainage stents in rabbit eyes *ex vivo* and *in vivo* ^23,24^. Other groups have previously used MRI to evaluate DDS behaviour in different settings. Kempe and colleagues described the use of a bench-top setup to investigate the formation and degradation of PLGA depots in the lower back area of mice^25^. Fernandez-Ferreiro *et al*. used MRI to monitor retention time of a possible drug delivery hydrogel on rat corneas, and Garcia *et al*. used MRI to assess polymer distribution after sub-tenon’s capsule injection in guinea pigs^26,27^.

In our current study, we started with *in vitro* experiments to assess the MRI properties of the matrix components separately and after polymerisation, using T2w sequences (Fig. 1). The hyaluronic acid in combination with latanoprost presented as a homogenously hyperintense mass with a few dark spots. These potentially resulted from small gas inclusions, which originated during loading of the syringe. ELA-NCO is strongly hypointense with no discernible signal in the used T2w MRI sequence. This is explained by the short T2-relaxation time of ELA-NCO, 19.7 ms, in comparison to the 296 ms of HA(LP), which accounts for the strong signal of hyaluronic acid with the used sequence parameters. The differences in T2-relaxation times of ELA-NCO and HA(LP) are largely influenced by the differences in water proton content of both components^12^. Whereas ELA-NCO does not contain water, HA(LP) was prepared as a 2.5% aqueous solution. Latanoprost, at the concentration used, does not have any measurable impact on the T2w signal of the HA(LP) suspension in our opinion. The change in distribution of hypo- and hyperintense areas between 10 minutes and 16 hours after mixing the components is partly due to the release of CO_2_ during the polymerisation process. The gas forms cavities which also appear hypointense in the used T2w sequence. Degradation of the depot *in vitro* via incubation in lysozyme containing buffer did not significantly change T2-relaxation times of the samples. This suggests that enzymatic cleavage of the 1,4-ß-glycosidic bonds between sugar monomers of the hyaluronic acid only results in an overall reduction of size and mass, without altering the chemical composition of the depot in a way that affects T2-relaxation. Therefore, the T2-relaxation time is not a suitable parameter to track degradation *in vitro*. SEM images revealed a similar surface structure of all degraded samples (Fig. 2). The varying sizes of the indentations seen in SEM images are attributed to chance and not considered to be caused by degradation, considering the wide range of pore size is likewise evident in the MR and micro-CT images. Analysis of overall sample porosity has been reported previously^12^.

*Ex vivo*, we imaged the drug depot at an in-plane resolution of 100 µm × 100 µm. It was easily distinguishable from surrounding ocular structures such as sclera, conjunctiva, and nictitating membrane. MRI and H&E stained section are comparable with regard to depot localisation (Fig. 3). The detailed visualisation of ocular tissues may suggest a potential of detecting possible inadvertent tissue reactions, such as fibrosis, *in vivo* using MRI and not only *post mortem* via H&E staining. Application of diffusion-weighted MRI to quantify renal fibrosis has been recently reported by Zhao and colleagues^28^.

*In vivo* MR scans showed the depot with nearly equal detail as *ex vivo* scans. The in-plane resolution for sagittal and coronal images was slightly lower than *ex vivo*, with 120 µm × 120 µm and 130 µm × 130 µm, respectively. In comparison to biomicroscopy, both methods complement each other, with the localisation more easily observed in biomicroscopy images, whereas the dimension for volume calculation of the drug depot within ocular tissues can only be precisely determined from MR images. Volumetric analysis was always done in sagittal sections, and for the first two time points quantifies the reduction of the depot volume accurately. At 21 weeks *in vivo* the drug depot was heavily fragmented, which might make volumetric estimation from MRI data less precise (Fig. 4b). The fragmentation *in vivo* is augmented by biochemical and cellular processes occurring at the site of implantation, such as release of degradation mediators, phagocytosis, and systemic resorption^29^. Fragmentation is possibly further aided by mechanical stress due to the movement of eyes and eye lids. It was not observed in the *in vitro* degraded samples. Although the volumetric quantification of the degradation process becomes more difficult for later time points, it is nonetheless possible and during the first 3 to 4 months after depot implantation it can provide reliable information regarding biodegradation. Photo documentation via a surgical microscope allows only visual investigation of the depot surface, this method, therefore, does not provide information about the volume or the bulk degradation of the depot. T2-relaxation studies to evaluate implant degradation were not performed *in vivo*. They would have induced further stress on the animals due to extended scan times, and *in vitro* data indicated that T2-relaxation time is not suitable to assess degradation of the DDS.

MRI also serves as a valuable tool to monitor ocular drug delivery and distribution^30^. Kim *et al*. have used MRI to monitor release of contrast agent from ocular implants^31,32^. Jockovich and colleagues used a 1.5 T system to observe drug localization and distribution after sub-tenon injections in NZW rabbits^33^. In future animal studies we will expand our MRI protocol to other aspects of the development process of DDS by assessing the suitability of a contrast agent such as gadolinium to monitor drug elution from the depot.

## Conclusion

In the present study, we demonstrated the feasibility of UHF-MRI as a non-invasive imaging tool for longitudinal evaluation of subconjunctival depot localisation in relation to other ocular tissues *in vivo*. We were able to quantify the biodegradation process of the depot in the same animal over several months. We achieved adequate image quality to distinguish the drug depot from the surrounding tissue at an in-plane resolution of >100 µm. The implementation of contrast agents into the DDS could potentially provide further information regarding drug release kinetics of periocular drug depots^30^.

Repeated measurements of the same animal reduce the number of required animals for a study, while scan times of less than 30 minutes minimise stress for the animals during individual measurements. Through the use of this imaging routine we promote the implementation of the 3R guidelines – reduce, refine, replace – for animal testing^34^.
